# Root-associated bacterial endophytes from *Ralstonia solanacearum* resistant and susceptible tomato cultivars and their pathogen antagonistic effects

**DOI:** 10.3389/fmicb.2015.00255

**Published:** 2015-04-14

**Authors:** Reshmi Upreti, Pious Thomas

**Affiliations:** Endophytic and Molecular Microbiology Laboratory, Division of Biotechnology, ICAR – Indian Institute of Horticultural ResearchBangalore, India

**Keywords:** 16S rRNA homology, bacterial wilt resistance, biological control, confocal microscopy, endophytic bacteria, *Ralstonia solanacearum* Solanum lycopersicum, tomato

## Abstract

This study was undertaken to assess if the root-associated native bacterial endophytes in tomato have any bearing in governing the host resistance to the wilt pathogen *Ralstonia solanacearum*. Internal colonization of roots by bacterial endophytes was confirmed through confocal imaging after SYTO-9 staining. Endophytes were isolated from surface-sterilized roots of 4-weeks-old seedlings of known wilt resistant (R) tomato cultivar Arka Abha and susceptible (S) cv. Arka Vikas on nutrient agar after plating the tissue homogenate. Arka Abha displayed more diversity with nine distinct organisms while Arka Vikas showed five species with two common organisms (*Pseudomonas oleovorans* and *Agrobacterium tumefaciens*). Screening for general indicators of biocontrol potential showed more isolates from Arka Abha positive for siderophore, HCN and antibiotic biosynthesis than from Arka Vikas. Direct challenge against the pathogen indicated strong antagonism by three Arka Abha isolates (*P. oleovorans, Pantoea ananatis,* and* Enterobacter cloacae*) and moderate activity by three others, while just one isolate from Arka Vikas (*P. oleovorans*) showed strong antagonism. Validation for the presence of bacterial endophytes on three R cultivars (Arka Alok, Arka Ananya, Arka Samrat) showed 8–9 antagonistic bacteria in them in comparison with four species in the three S cultivars (Arka Ashish, Arka Meghali, Arka Saurabhav). Altogether 34 isolates belonging to five classes, 16 genera and 27 species with 23 of them exhibiting pathogen antagonism were isolated from the four R cultivars against 17 isolates under three classes, seven genera and 13 species from the four S cultivars with eight isolates displaying antagonistic effects. The prevalence of higher endophytic bacterial diversity and more antagonistic organisms associated with the seedling roots of resistant cultivars over susceptible genotypes suggest a possible role by the root-associated endophytes in natural defense against the pathogen.

## Introduction

Endophytic microorganisms colonize plants internally without any apparent adverse effects on the host (Hallmann et al., 1997; Gaiero et al., 2013). There is a growing interest in endophytic bacteria on account of their potential use in plant growth promotion, antagonistic effect on pests and pathogens, alleviation of abiotic stress and in phytoremediation (Compant et al., 2005; Ryan et al., 2008; Mercado-Blanco and Lugtenberg, 2014). Bacterial endophytes are generally known to enter the host from the surrounding soil through wounds in the roots (Hallmann et al., 1997; Compant et al., 2010) or through root hairs (Prieto et al., 2011; Mercado-Blanco and Prieto, 2012). They traverse the root cortex and reach various plant organs through the vascular system (Hallmann et al., 1997; Compant et al., 2010, 2011) while some use the apoplastic route (Sattelmacher, 2001; Reinhold-Hurek et al., 2007). Bacterial endophytes were earlier considered to be primarily colonizers in the inter-cellular or apoplastic spaces in the roots being present in relatively fewer numbers (Hallmann et al., 1997; Hallmann, 2001). Molecular studies have shown that there is considerable species diversity of bacterial endophytes albeit being present largely in a non-cultivable form (Lundberg et al., 2012; Sessitsch et al., 2012; Podolich et al., 2015). Intracellular colonization has also been documented in some plant systems (Pirttilä et al., 2000; de Almeida et al., 2009). A recent study employing banana shoot tissue has shown abundant endophytic bacteria in the two intracellular niches, namely in the cytoplasm and in the perispace between the cell wall and plasma membrane, and the terms ‘Cytobacts’ and ‘Peribacts’ have been coined to recognize the microorganisms in the respective intracellular niches (Thomas and Reddy, 2013; Thomas and Sekhar, 2014).

Bacterial wilt caused by the vascular pathogen, *Ralstonia solanacearum* (syn. *Pseudomonas solanacearum*) is a major constraint for tomato cultivation world over (Hayward, 1991; Genin and Denny, 2012). The wide host range covering major food and other economically important crops, broad geographic distribution, adaptation to survive in soil and water for long periods and the huge economic loss incited make the pathogen a very significant one worldwide (Genin and Denny, 2012; Mansfield et al., 2012). *R. solanacearum* invades the host through root injuries. The pathogen crosses the root cortex and overruns the xylem vessels leading to sudden wilting and plant death (Hayward, 1991; Genin and Denny, 2012). The similarities between bacterial endophytes and *R. solanacearum* in xylem colonization render the former as potential antagonistic and biocontrol agents against such vascular pathogens (Achari and Ramesh, 2014; Ting, 2014). Use of antagonistic bacteria for the biocontrol of bacterial wilt in tomato has been documented either as rhizospheric organisms (Vanitha et al., 2009) or as endophytes isolated from the same crop (Feng et al., 2013) or unrelated crops (Thomas and Upreti, 2014a).

Endophytic bacteria share an intimate symbiotic association with the host which makes them more valuable biocontrol agents (Compant et al., 2005; Bakker et al., 2013). Endophytes get an edge over their rhizospheric antagonist-counterparts on account of their ability to enter the host system without stimulating pathogen induced vulnerability responses but triggering host defense pathways (Conn et al., 2008; Gómez-Lama Cabanás et al., 2014; Podolich et al., 2015). Being internal colonizers, they could provide a barrier against the invading pathogens directly or through the production of bio-active compounds (Thomas and Upreti, 2014a; Podolich et al., 2015). Endophytes are better protected against abiotic stress and competing microbes compared with the rhizospheric counterparts (Hallmann et al., 1997; Ryan et al., 2008; Turner et al., 2013). While a vast majority of bacterial endophytes are known to be non-amenable for cultivation on common media (Lundberg et al., 2012; Sessitsch et al., 2012; Thomas and Sekhar, 2014), it entails that the organisms are easily cultivated to allow their agricultural exploitations. The present study was undertaken with a view to explore the extent of cultivable endophytic bacteria in transplantable-stage seedling roots of tomato cultivars that are either resistant or susceptible to *R. solanacearum*. Further, it was envisaged to evaluate the antagonistic and biocontrol features of the isolates to determine if the native endophytes played any role in governing the resilient property of the resistant cultivars.

## Materials and Methods

### Plant Material

*Ralstonia solanacearum* resistant (R) tomato (*Solanum lycopersicum* L.) cultivar Arka Abha and susceptible (S) cv. Arka Vikas (Thomas et al., 2015) were taken up as the primary test material in this study. In order to validate the findings, additional resistant (Arka Alok, Arka Ananya)/moderately resistant (Arka Samrat) and susceptible (Arka Ashish, Arka Meghali, and Arka Saurabhav) cultivars were employed. The names of genotypes are prefixed with R, MR, or S for easy recognition as resistant, moderately resistant or susceptible, respectively. Seedlings were raised in pasteurized organic cocopeat in protrays (Thomas et al., 2015) and used for the isolation of endophytes after 3½–4 weeks which corresponded to the stage of transplanting to the field when seedlings normally get exposed to the field pathogen inoculum (Thomas and Upreti, 2014b).

### Confocal Imaging of Seedling Roots

Seedling roots were examined for bacterial colonization through confocal laser scanning microscopy (CLSM) after SYTO-9 staining. For this, tender roots from 3 to 4 weeks-old cocopeat – grown seedlings were washed, cut to ∼1 cm segments and were treated with 1× SYTO-9 (12 μM) from the LIVE/DEAD BacLight^®^ bacterial viability kit L13152 (Molecular Probes, Invitrogen) as per the kit instructions. After 10–15 min staining, the lateral roots and root hairs were examined using a LSM 5 LIVE confocal microscope and the images were processed as described elsewhere (Thomas and Reddy, 2013). Root tissues were also examined after surface sterilization which involved a quick dip in 90% ethanol, a rinse in sterile distilled water (SDW) and 1 min sodium hypochlorite (2% available chlorine) treatment followed by six SDW rinses.

### Isolation of Endophytes from Seedling Roots

Twenty randomly picked seedlings from ^R^Arka Abha and ^S^Arka Vikas 4 weeks after sowing were lifted with the plug of cocopeat and washed under running water taking care to minimize root injury. Seedlings were excised below the cotyledonary node and surface-sterilized essentially as per Zinniel et al. (2002). This involved a quick dip in 90% ethanol, a rinse in SDW and 1 min NaOCl (2% chlorine) treatment as above. After three rinses in SDW, 2% Na_2_S_2_O_3_ (10 min) was used to remove chloramine residues before finally rinsing the roots in SDW thrice. Root part was excised, blotted dry, weighed aseptically and macerated in a mortar employing 12.5 mM potassium phosphate buffer (Zinniel et al., 2002). After adjusting the volume to 10 ml g^-1^ tissue weight (10^0^ stock), serial dilutions (10^1^–10^5^) were applied on NA through spotting- and tilt-spreading (SATS) approach (Thomas et al., 2012) with three replications per dilution. The plates were incubated at 30^∘^C and the colony forming units (cfu) g^-1^ root tissue was determined on the third day. The NA plates used in this study were pre-monitored for absolute microbial sterility.

### Identification of Organisms

Distinct bacterial colony types that emerged on NA from the root homogenate of ^R^Arka Abha (Tm-Ab01 to Tm-Ab09) and ^s^Arka Vikas (Tm-Av01 to Av05), serially numbered in the order of their relative abundance, were further purified through three rounds of streaking on NA. They were identified through partial 16S rRNA sequence homology analysis. For this polymerase chain reaction (PCR) was carried out with the primers 27F (5′-AGAGTTTGATCCTGGCTCAG-3′) and 1492R-Y (5′-GGYTACCTTGTTACGACTT-3′; Y = C/T) with the thermocyling conditions as described elsewhere (Thomas et al., 2008). The identity of these organisms was established and validated through megablast analysis to the cultured organisms at the National Centre for Biotechnological Information (NCBI) and the Seqmatch analysis with the Type Strains at the Ribosomal Database Project (RDP), Michigan State University. Wherever the identification was inconclusive based on NCBI homologies in the case of less common organisms, the highest species homology from NCBI or the similarity score from RDP was adopted to suggest the identity at sequence data submission to NCBI. The final identity was fixed as per the genus/species assigned by the GenBank at the acceptance of sequence data.

### Screening of Organisms for the Indicators of Biocontrol Property

The endophytic organisms were tested for siderophore production through chrome azurol S method (Schwyn and Neilands, 1987) and for HCN production as per Ahmad et al. (2008). The isolates were screened through PCR for functional genes involved in the biosynthesis of bacterial non-ribosomal peptide synthetase (NRPS) and polyketide synthase (PKS) as markers for antibiotic production as per Miller et al. (2012). The primers MTF2 (5′-GCNGGYGGYGCNTAYGTNCC-3′) and MTR2 (5′-CCNCGDAYTTNACYTG-3′) were employed for NRPS giving a PCR product of ∼1000 bp, and the primers DKF (5′-GTGCCGGTNCCRTGNGYYTC-3′) and DKR (5′-GCGATGGAYCCNCARCARMG-3′) for PKS yielding ∼650–700 bp PCR product.

### Pathogen and Culture Media

*Ralstonia solanacearum* ‘NH-Av01’ strain (NCBI acc. no. KJ412034; biovar 3) isolated from the bacterial ooze of a wilted ‘Arka Vikas’ plant as described elsewhere (Thomas and Upreti, 2014b,c) was used in antagonistic assays. The culture was stored as glycerol stocks at –80^∘^C and revived on Kelman (1954) medium containing 1.0 g l^-1^ casein hydrolysate (C), 10 g l^-1^ bacteriological peptone (P), 5 g l^-1^ glucose (G), and 15 g l^-1^ bacteriological agar (A) and was fortified with 0.005% 2,3,5-Triphenyltetrazolium chloride (KM-TTC). The media constitutes were sourced from Hi Media Biosciences, Mumbai, except for TTC (Sigma, St. Louis, MO, USA) employing P14 lot of Type-1 peptone as per Thomas and Upreti (2014c). This was based on the observation that the colony characteristics, lawn formation and inhibition zone development were significantly influenced by the type and batch of peptone. Other media employed included casein-peptone-glucose-agar (CPGA) or CPG broth. Three additional *Ralstonia* isolates, namely, NH-Av05, NH-Av07, and KAU-Av01 were also used in the antagonistic assays.

### Antagonistic Assay

Antagonistic assays were set up essentially as described earlier (Thomas and Upreti, 2014a). Briefly, 200 μl of 2-days-old CPGA or KM-TTC culture of 0.1 OD at 600 nm (approximately cfu of 10^8^ ml^-1^) in peptone – salt (1 g l^-1^ each peptone and NaCl; Thomas et al., 2012) was spread over KM-TTC medium in 12 cm × 12 cm plates (Hi Media Biosciences, Mumbai) and wells of 6–7 mm diameter were prepared. After allowing *R. solanacearum* to establish at 30^∘^C for 4 h, 50 μl of 0.2 OD endophytic bacterial inoculums in peptone – salt (approximately cfu in the range of 10^7^–10^8^ ml^-1^ for 0.1 OD culture depending on the organism) was applied in marked wells. After 20–25 min of surface drying, the plates were incubated inverted at 30^∘^C. The antagonistic potential was rated based on the extent of clear zone formation, namely, strong (>20 mm; +++), medium (15–20 mm; ++), low (10–15 mm; +), or none.

### Validation with Additional Tomato Cultivars

This included three additional resistant cultivars/F1 hybrids (^R^Arka Alok, ^R^Arka Ananya F1, ^MR^Arka Samrat F1) and three susceptible cultivars (^S^Arka Ashish, ^S^Arka Meghali, ^S^Arka Saurabhav; Thomas et al., 2015). Seedlings were grown in cocopeat in protrays and 5–10 surface-sterilized seedlings at 3½–4 weeks stage were employed for isolating the root endophytes. Tissue processing, culture purification, identification and assay for the antagonistic potential against the pathogen were undertaken as described earlier.

### Nucleotide Sequences

The partial 16SrRNA gene sequences of the organisms have been deposited with the NCBI GenBank. The accession numbers are indicated in the Tables describing their identification.

## Results

### Confocal Imaging of Seedling Roots

**FIGURE 1:**
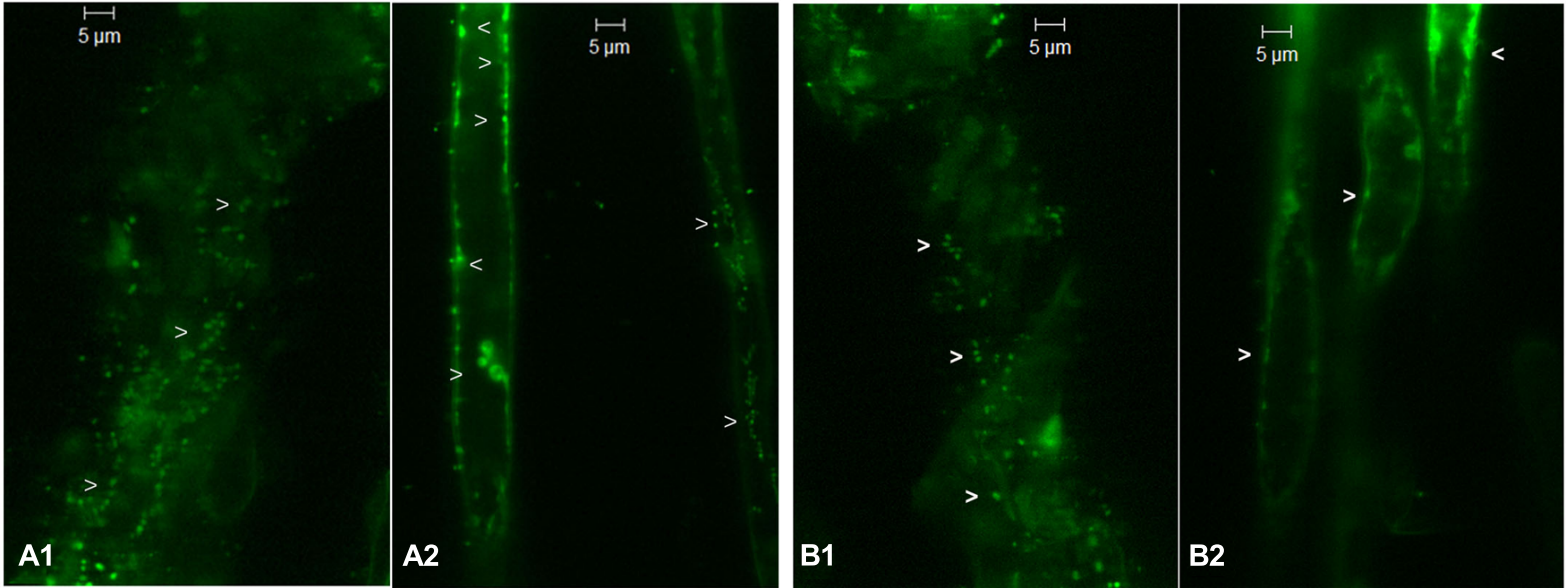
Confocal laser scanning microscopy images from SYTO-9 treated non-surface sterilized roots of tomato ^S^Arka Vikas and ^R^Arka Abha showing green fluorescing bacteria (indicated by arrow heads) on the surface (**A1,B1**, respectively) and internally along the cell periphery and inside root hairs (**A2,B2**, respectively).

The tender roots from 3 to 4 weeks-old ^R^Arka Abha and ^S^Arka Vikas seedlings showed green fluorescing bacterial cells on the root surface, inside the roots and in the surrounding film of water after SYTO-9 staining (**Figures 1A1,B1**). Root hairs showed abundant bacteria internally both along the cell periphery and in the cytoplasm (**Figures 1A2,B2**) confirming the endophytic colonization. Following surface sterilization, confocal imaging was impaired due to rapid signal bleaching (data not shown). However, it was possible to track the bacterial cells in both tender roots and root hairs with a notable reduction in the counts.

### Isolation and Identification of Endophytes from ^R^Arka Abha and ^S^Arka Vikas

Root growth in ^R^Arka Abha seedlings at endophyte isolation stage was relatively low compared with ^S^Arka Vikas. However, both the genotypes showed similar cfu estimates per unit fresh tissue weight (3.9 × 10^4^ and 4.3 × 10^4^, respectively). A number of distinct colonies were picked up which were finally assigned to nine distinct species in ^R^Arka Abha and five species in ^S^Arka Vikas (**Table 1**). The organisms from ^R^Arka Abha as per 16S rRNA gene sequence data accepted at NCBI GenBank included *Pseudomonas oleovorans*, *Pseudomonas plecoglossicida, Pantoea ananatis*, *Citrobacter freundii*, *Staphylococcus hominis*, *Sphingobacterium multivorum*, *Enterobacter cloacae*, *Arthrobacter globiformis,* and *Agrobacterium tumefaciens*. The isolates from ^S^Arka Vikas constituted *P. oleovorans*, *Stenotrophomonas maltophilia*, *Bacillus pumilus, A. tumefaciens,* and *Microbacterium pumilum*. The resistant cultivar apparently displayed more endophytic bacterial diversity with two organisms (*P. oleovorans* and *A. tumefaciens*) common to both the cultivars. Both ^R^Arka Abha and ^S^Arka Vikas showed more of Gram-negative bacteria (78 and 60%, respectively) and γ-subclass of Proteobacterium formed the commonest single phylogenetic group in both the cultivars (56 and 40%, respectively).

**Table 1:** Identification of bacterial endophytes isolated from the seedling root tissue of tomato cvs. Arka Abha and Arka Vikas.

No.	Isolate ID	16S seq (bp) and NCBI acc. No	Identity based on closest species from NCBI/RDP (with acc. no and homology/similarity score)^†^	Phylogenic group and Gram reaction
**Isolates from resistant cv. Arka Abha**
1	Tm- Ab01	770 (KM349750)	*Pseudomonas oleovorans * (HQ697330; 99%)	γ-Proteobacterium; -ve
2	Tm- Ab02	767 (KM349751)	*Pseudomonas plecoglossicida* (KJ395363; 99%)	γ-Proteobacterium; -ve
3	Tm- Ab03	711 (KM349752)	*Pantoea ananatis* (HQ683996; 98%)	γ-Proteobacterium; -ve
4	Tm- Ab04	793 (KM349753)	*Citrobacter freundii* (KF769539; 99%)	γ-Proteobacterium; -ve
5	Tm- Ab05	777 (KM349754)	*Staphylococcus hominis* (KJ018991; 100%)	Firmicute; +ve
6	Tm- Ab06	856 (KM349755)	*Sphingobacterium multivorum* (KF535161; 99%)	Bacteroidetes; -ve
7	Tm- Ab07	951 (KM349756)	*Enterobacter cloacae* (KF971358; 99%)	γ-Proteobacterium; -ve
8	Tm- Ab08	725 (KM349757	*Arthrobacter globiformis* (KJ124593; 99%)	Actinobacterium; -ve
9	Tm- Ab09	750 (KM349758)	*Rhizobium radiobacter * (S000721046; 0.967)^#^NCBI: *Agrobacterium tumefaciens*	α-Proteobacterium; -ve
**Isolates from susceptible cv. Arka Vikas**
1	Tm-Av01	794 (KM349745)	*Pseudomonas oleovorans* (HQ697330; 99%)	γ-Proteobacterium; -ve
2	Tm-Av02	860 (KM349746)	*Stenotrophomonas maltophilia* (KM108534; 99%)	γ-Proteobacterium; -ve
3	Tm-Av03	810 (KM349747)	*Bacillus pumilus* (KC834607; 100%)	Firmicute; +ve
4	Tm-Av04	818 (KM349749)	*Rhizobium radiobacter * (S000721046; 1.0)^#^NCBI:* Agrobacterium tumefaciens*	α-Proteobacterium; -ve
5	Tm-Av05	662 (KM349750)	*Microbacterium pumilum* (KC213957; 99%)	Actinobacterium; +ve

*^†^As on 20 August 2014 at sequence submission to NCBI GenBank.*

*#Identity assigned by NCBI GenBank.*

### Assessing the Endophytes for the Indicators of Biocontrol Property

Two of the ^R^Arka Abha isolates (Tm-Ab01, Tm-Ab03) showed siderophore production, two isolates (Tm-Ab03, Tm-Ab07) HCN production and three isolates (Tm-Ab02, Tm-Ab06, Tm-Ab08) proved positive for NRPS/ PKS (**Table 2**). The respective numbers for ^S^Arka Vikas were one, zero and one. Thus, the resistant cultivar harbored more organisms with biocontrol properties than the susceptible cultivar.

**Table 2:** Screening of bacterial endophytes from* Ralstonia* resistant Arka Abha and susceptible Arka Vikas tomato cultivars for the indicators of bio-control property.

Isolate	Endophytic organism	Bio-control property indicator				Extent of inhibition zone
		Siderophore	HCN	Antibiotic markers		
				NRPS	PKS	
**Isolates from resistant cv. Arka Abha**
Tm-Ab01	*Pseudomonas oleovorans*	×	_	_	_	+++
Tm-Ab02	*Pseudomonas plecoglossicida*	_	_	_	×	++
Tm-Ab03	*Pantoea ananatis*	×	×	_	_	+++
Tm-Ab04	*Citrobacter freundii*	_	_	_	_	-
Tm-Ab05	*Staphylococcus hominis*	_	_	_	_	+
Tm-Ab06	*Sphingobacterium multivorum*	_	_	×	_	+
Tm-Ab07	*Enterobacter cloacae*	_	×	_	_	+++
Tm-Ab08	*Arthrobacter globiformis*	_	_	×	_	++
Tm-Ab09	*Agrobacterium tumefaciens*	_	_	_	_	-
**Isolates from susceptible cv. Arka Vikas**
Tm-Av01	*Pseudomonas oleovorans*	×	_	_	_	+++
Tm-Av02	*Stenotrophomonas maltophilia*	_	_	_	_	+
Tm-Av03	*Bacillus pumilus*	_	_	×	_	+
Tm-Av04	*Agrobacterium tumefaciens*	_	_	_	_	-
Tm-Av05	*Microbacterium pumilum*	_	_	_	_	-

*_, Negative; × , positive; Antagonistic activity: none (-), low (+), medium (++), or high (+++).*

### Screening of Endophytes for *Ralstonia* Antagonistic Activity

**FIGURE 2:**
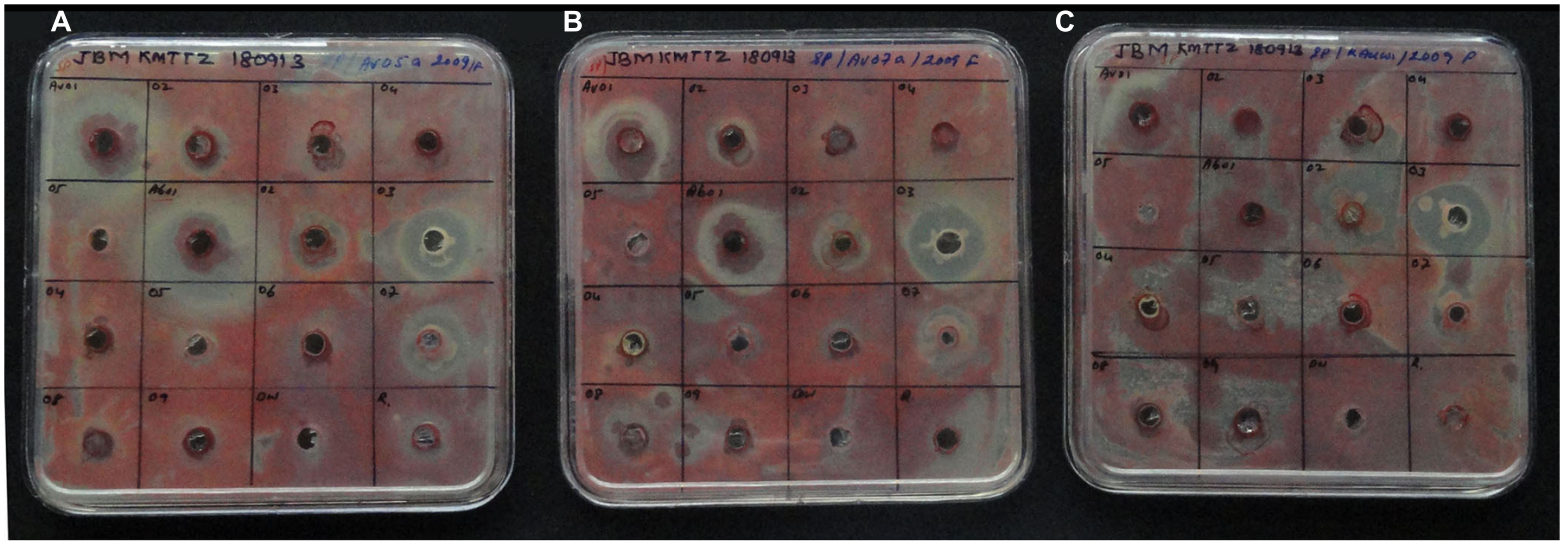
Screening of bacterial endophytes from susceptible cv. Arka Vikas and resistant cv. Arka Abha for the antagonistic activity against *Ralstonia solanacearum* isolates NH-Av05 **(A)**, NH-Av07 **(B)**, and KAU-Av01 **(C)**. Treatment order: Row 1: Tm-Av01 to Av04; Row 2: Tm-Av05, Tm-Ab01 to Ab03; Row 3: Tm-Ab04 to Ab07; Row 4: Tm-Ab08, Ab09, distilled water control, *Ralstonia* inoculum, respectively.

Seven isolates from ^R^Arka Abha showed varying extents of antagonistic activity against *R. solanacearum* with Tm-Ab01 (*P. oleovorans*), Tm-Ab03 (*P. ananatis*), and Tm-Ab07 (*E. cloacae*) displaying significant effects, two isolates (Tm-Ab02, Tm-Ab08) offering medium activity and two others (Tm-Ab05, Tm-Ab06) showing low activity (**Table 2**). Among the ^S^Arka Vikas isolates, Tm-Av01 (*P. oleovorans*) showed strong antagonism while Tm-Av02 and Tm-Av03 displayed low activity. This was found true in a repeat assay and with three other isolates of *R. solanacearum*, namely, NH-Av05, NH-Av07 and KAU-Av01 (**Figure 2**).

### Validation with Additional Resistant and Susceptible Cultivars

^R^Arka Alok, ^R^Arka Ananya, and ^MR^Arka Samrat yielded 8–9 distinct organisms each while ^S^Arka Ashish, ^S^Arka Meghali, and ^S^Arka Saurabhav gave rise to four species each constituting a total of 37 isolates (**Table 3**). In general, there was a predominance of Gram negative bacteria in four cultivars (78, 62.5, 75, and 75%, respectively in ^R^Arka Alok, ^R^Arka Ananya, ^S^Arka Ashish, and ^S^Arka Saurabhav). However, ^MR^Arka Samrat and ^S^Arka Meghali showed 88 and 50% Gram positive organisms, respectively. The resistant cultivars showed more organisms with antagonistic potential in comparison with susceptible cultivars (**Table 3**) as discussed below.

**Table 3:** Identification of bacterial endophytes from additional resistant and susceptible cultivars and their antagonistic activity against *Ralstonia solanacearum* NH-Av01 determined through agar-well diffusion assay.

Isolate	16S seq (bp) and NCBI acc. no	Identity based on closest species from NCBI/RDP (with acc. no and homology/similarity score)^†^	Phylogenic group and Gram reaction	Antagonistic effect
**Arka Alok (Resistant) 6 × 10^**5**^ cfu g^**->1**^ (nine isolates)**
Tm-Alk01	910 (KM603626)	*Bacillus megaterium * (KJ789369; 99%)	Firmicute; +ve	+
Tm-Alk02	822 (KM603627)	*Asticcacaulis benevestitus * (S000592821; 0.798)	α-Proteobacteria; -ve	+
Tm-Alk03	850 (KM603628)	*Microbacterium oleivorans * (KF307652; 99%)	Actinobacteria; +ve	-
Tm-Alk04	914 (KM603629)	*Hydrogenophaga intermedia * (FJ009392; 99%)	β-Proteobacteria; -ve	-
Tm-Alk05	892 (KM603630)	*Novosphingobium subterraneum* (FJ527720; 99%) ^#^*Novosphingobium aromaticivorans*	α-Proteobacteria; -ve	+
Tm-Alk06	700 (KM603631)	*Pantoea ananatis* (HE716948; 98%)	γ-Proteobacteria; -ve	+
Tm-Alk07	950 (KM603632)	*Enterobacter cloacae* (KM077045; 99%)	γ-Proteobacteria; -ve	+++
Tm-Alk08	725 (KM603633)	*Pseudomonas taiwanensis * (S001095516; 0.918)	γ-Proteobacteria; -ve	+
Tm-Alk09	575 (KM603634)	*Pseudomonas otitidis * (KF699886; 99%)	γ-Proteobacteria; -ve	++
**Arka Ananya (Resistant) 6 × 10^**4**^ cfu g^**-1**^ (eight isolates)**				
Tm-Ana01	750 (KM603635)	*Enterobacter ludwigii* (S000539659; 0.972)	γ-Proteobacteria; -ve	++
Tm-Ana02	925 (KM603636)	*Bacillus megaterium * (KJ789369; 99%)	Firmicute; +ve	-
Tm-Ana03	870 (KM603637)	*Chryseobacterium taiwanense* (KC122691; 99%)	Flavobacteria; -ve	-
Tm-Ana04	900 (KM603638)	*Rhizobium oryzae* (S001168838; 0.846)	α-Proteobacteria; -ve	+
Tm-Ana05	770 (KM603639)	*Staphylococcus hominis* (KJ197177; 99%)	Firmicute; +ve	+
Tm-Ana06	780 (KM603640)	*Pseudomonas otitidis * (LN558646; 99%)	γ-Proteobacteria; -ve	++
Tm-Ana07	900 (KM603641)	*Staphylococcus haemolyticus* (HG941667; 99%)	Firmicute; +ve	+++
Tm-Ana08	720 (KM603642)	*Pseudomonas taiwanensis* (S001095516; 0.918)	γ-Proteobacteria; -ve	+
**Arka Samrat (Moderately resistant) 4.7 × 10^**3**^ cfu g^**-1**^ (eight isolates)**				
Tm-Sam01	920 (KM603643)	*Microbacterium lacticum * (S000013457; 0.947)	Actinobacteria; +ve	-
Tm-Sam02	895 (KM603644)	*Bacillus megaterium * (KF381342; 99%)	Firmicute; +ve	+
Tm-Sam03	555 (KM603645)	*Microbacterium pumilum* (LK391536; 99%)	Actinobacteria; +ve	-
Tm-Sam04	890 (KM603646)	*Bacillus safensis* (S000458519; 0.996)	Firmicute; +ve	+
Tm-Sam05	975 (KM603647)	*Bacillus soli* (S000323282; 0.948)	Firmicute; +ve	-+
Tm-Sam06	915 (KM603648)	*Bacillus bataviensis * (S000323277; 0.933)	Firmicute; +ve	-
Tm-Sam07	810 (KM603649)	*Corynebacterium amycolatum* (KF539917; 99%)	Actinobacteria; +ve	-
Tm-Sam 08	850 (KM603650)	*Rhizobium radiobacter * (S000721046; 0.987)^#^*Agrobacterium tumefaciens*	α-Proteobacteria; -ve	-
**Arka Ashish (Susceptible) 1.9 × 10^4^ cfu g^-**1**^ (four isolates)**
Tm-Ash01	550 (KM603651)	*Microbacterium oleivorans* (KF777385; 99%)	Actinobacteria; +ve	-
Tm-Ash02	910 (KM603652)	*Pseudoxanthomonas mexicana* (KF358265; 99%)	γ-Proteobacteria; -ve	+
Tm-Ash03	905 (KM603653)	*Rhizobium pseudoryzae * (S002221791; 0.913)	α-Proteobacteria; -ve	-
Tm-Ash04	930 (KM603654)	*Acidovorax soli * (S001293324; 0.937)	β-Proteobacteria; -ve	-
**Arka Meghali (Susceptible) 3.1 × 10^**4**^ cfu g^**-1**^ (four isolates)**
Tm-Meg 01	968 (KM603655)	*Pseudomonas otitidis* (KF668329; 100%)	γ-Proteobacteria; -ve	+
Tm-Meg 02	690 (KM603656)	*Microbacterium oleivorans* (KF777385; 100%)	Actinobacteria; +ve	-
Tm-Meg 03	908 (KM603657)	*Bacillus megaterium * (S000979521; 0.961)	Firmicutes; +ve	+
Tm-Meg 04	865 (KM603658)	*Asticcacaulis benevestitus* (S000592821; 0.796)	α-Proteobacteria; -ve	-
**Arka Saurabhav (Susceptible) 6.5 × 10^**4**^ cfu g^**-1**^ (four isolates)**
Tm-Sau01	680 (KM603659)	*Microbacterium oleivorans * (KF777385; 100%)	Actinobacteria; +ve	-
Tm-Sau02	795 (KM603659)	*Pseudoxanthomonas mexicana* (KF135463; 99%)	γ-Proteobacteria; -ve	-
Tm-Sau03	905 (KM603661)	*Pseudomonas alcaliphila* (KC699534; 99%)	γ-Proteobacteria; -ve	+
Tm-Sau04	855 (KM603662)	*Acidovorax soli * (S001293324; 0.934)	β-Proteobacteria; -ve	-

*^†^As on September 2014 at NCBI Submission.*

*^†^Identity assigned by NCBI GenBank at sequence acceptance.*

*Antagonistic activity: low (+), medium (++), or high (+++).*

### Endophytes in Resistant and Susceptible Cultivars in Relation to Pathogen Antagonism

**FIGURE 3:**
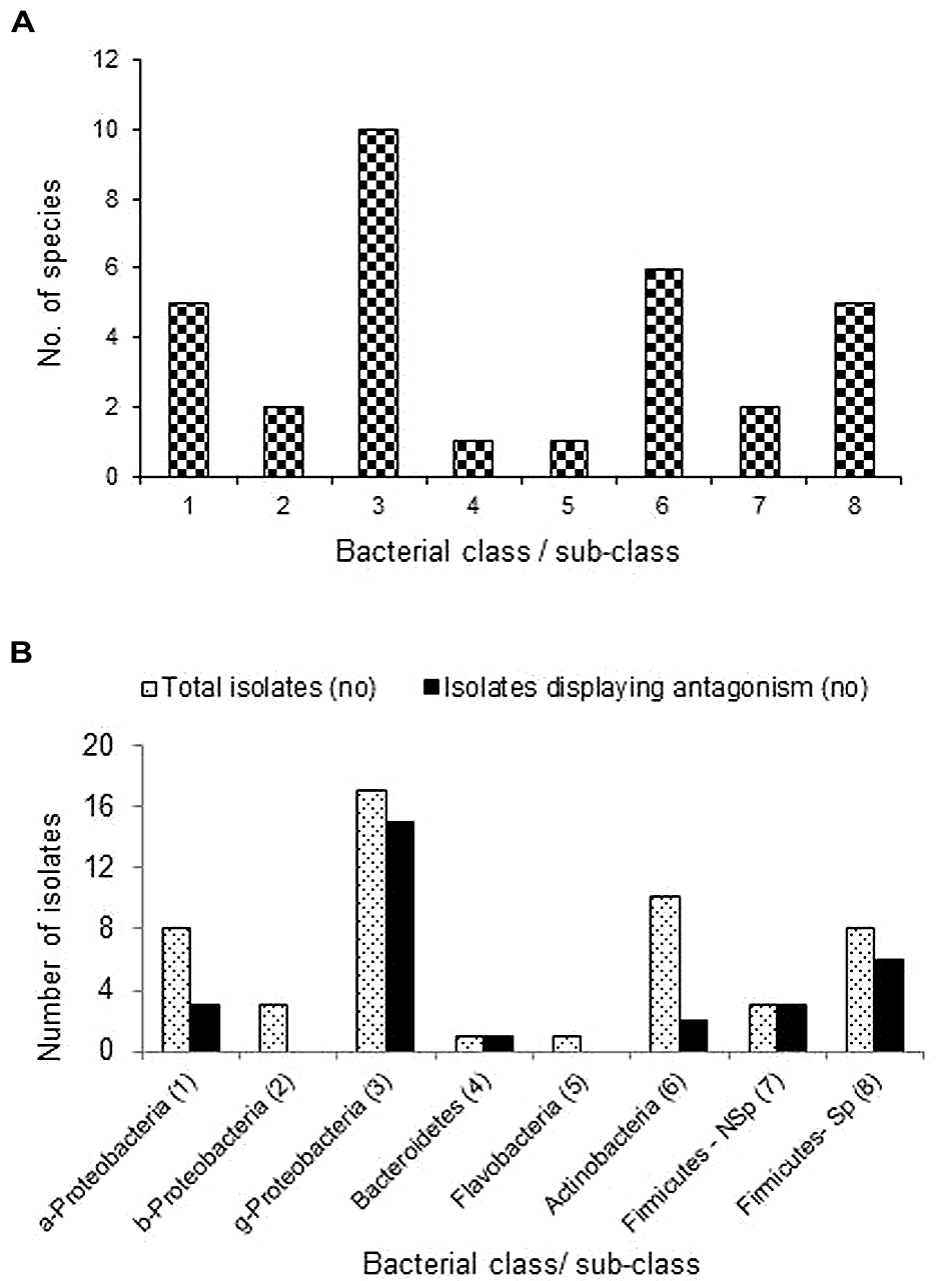
Extent of cultivable bacterial endophytes belonging to different bacterial classes and sub-classes across four *Ralstonia* resistant and four susceptible tomato cultivars **(A)**, and the extent of organisms belonging to different phylogenetic groups displaying antagonistic activity against the pathogen **(B)**; a-, b-, g- Proteobacteria represents α, β, and γ sub-classes; Firmicutes-Nsp and Sp represent non-sporulating and spore-forming Firmicutes.

When the whole spectrum of root-associated bacterial endophytes in the four resistant and four susceptible cultivars of this investigation is considered, γ-Proteobacteria formed the commonest group followed by Actinobacteria, α-Proteobacteria and spore-forming Firmicutes (**Figure 3A**). The four resistant cultivars together yielded 34 endophytic bacteria which belonged to five classes (Proteobacteria, Actinobacteria, Firmicutes, Bacteroidetes, and Flavobacteria), 16 genera and 27 species while the isolates from susceptible cultivars represented three classes (Proteobacteria, Actinobacteria, and Firmicutes) including seven genera and 13 species (**Table 4**). The number of organisms displaying antagonistic activity during agar-well diffusion assay ranged from 4 to 7 in the former group while it was only one or two in the latter. Thus, among the R-cultivar isolates, 23 of them displayed varying levels of antagonistic effects while just seven from the S- category displayed such responses. Further, the extent of antagonistic activity as indicated by the diameter of clear zone was more with the isolates from R sources which included *P. oleovorans, P. ananatis,* and *E. cloacae* from ^R^Arka Abha,* E. cloacae* and* P. otitidis* from ^R^Arka Alok, and *E. ludwigii, P. otitidis,* and *Staphylococcus haemolyticus* from ^R^Arka Ananya. Maximum organisms with the antagonistic activity was observed with the γ-Proteobacteria group constituted by the genera *Enterobacter, Pseudomonas,* and *Pantoea* spp. with 15 out of 17 isolates showing antagonistic effects (**Figure 3B**). The next most promising group included non-spore forming Firmicutes, namely *S. haemolyticus* and *S. hominis* with all three isolates displaying good antagonistic potential.

**Table 4:** Extent of diversity of endophytic bacteria in *Ralstonia* resistant and susceptible cultivars of tomato in relation to pathogen antagonistic effect.

S. no	Phylogenetic group	Resistant cultivars				Susceptible cultivars			
		Arka Abha	Arka Alok	Arka Ananya	Arka Samrat^†^	Arka Vikas	Arka Ashih	Arka Meghali	Arka Saurabhav
	**α-Proteobacteria**
1	*Asticcacaulis benevestitus*		•/+					•/-	
2	*Agrobacterium tumefaciens*	•/-			•/-	•/-			
3	*Rhizobium oryzae*			•/-					
4	*Rhizobium pseudoryzae*						•/+		
5	*Novosphingobium aromaticivorans*		•/+						
	**β-Proteobacteria**
6	*Acidovorax soli*						•/-		•/-
7	*Hydrogenophaga intermedia*		•/-						
	**γ-Proteobacteria**
8	*Enterobacter cloacae*	•/+++	•/+++						
9	*Enterobacter ludwigii*			•/++					
10	*Pseudomonas alcaliphila*								•/+
11	*Pseudomonas oleovorans*	•/+++				•/+++			
12	*Pseudomonas otitidis*		•/++	•/++				•/+	
13	*Pseudomonas plecoglossicida*	•/+							
14	*Pseudomonas taiwanensis*		•/+	•/+					
15	*Pseudoxanthomonas mexicana*						•/-		•/-
16	*Pantoea ananatis*	•/+++	•/+						
17	*Stenotrophomonas maltophilia*					•/+			
	**Bacteroidetes**
18	*Sphingobacterium multivorum*	•/+							
	**Flavobacteria**
19	*Chryseobacterium taiwanense*			•/-					
	**Actinobacteria**
20	*Arthrobacter globiformis*	•/+							
21	*Citrobacter freundii*	•/-							
22	*Corynebacterium amycolatum*				•/+				
23	*Microbacterium lacticum*				•/-				
24	*Microbacterium oleivorans*		•/-				•/-	•/-	•/-
25	*Microbacterium pumilum*				•/-	•/-			
	**Firmicutes – non-sporulating**
26	*Staphylococcus haemolyticus*			•/+++					
27	*Staphylococcus hominis*	•/+		•/+					
	**Firmicutes – sporulating**
28	*Bacillus bataviensis*				•/-				
29	*Bacillus pumilus*					•/+			
30	*Bacillus safensis*				•/+				
31	*Bacillus megaterium*		•/+	•/-	•/+			•/+	
32	*Bacillus soli*		•/+	•/-	•/+			•/+	
	Isolates showing antagonistic effect/Total	7/9	7/9	5/8	4/8	3/5	1/4	2/4	1/4

*•, Presence in the cultivar; –, no antagonistic activity; +, ++, +++: low, medium, or strong Ralstonia solanacearum antagonistic activity, respectively.*

## Discussion

Bacterial endophytes are known to confer protection against pathogens in a number of diseases (Compant et al., 2005; Mercado-Blanco and Lugtenberg, 2014) including *Ralstonia* wilt in tomato (Tan et al., 2011; Feng et al., 2013) and in related solanaceous crops (Ramesh and Phadke, 2012; Achari and Ramesh, 2014). Not many studies have addressed the diversity of endophytes or their possible involvement in offering a natural protection against this pathogen. The present study covering a number of tomato cultivars belonging to the resistant and susceptible categories enunciated the presence of greater cultivable endophytic bacterial diversity and more organisms with pathogen antagonistic potential in resistant cultivars. The isolates with antagonistic potential from resistant cultivars often showed accentuated pathogen inhibitory activity with one exception of Arka Samrat, which belonged to the moderately resistant category (Thomas et al., 2015). These observations suggested the possibility of an active role played by the endophytes in providing a natural protection against the pathogen in resistant cultivars. A recent study in tomato involving just one cultivar each from *Ralstonia* resistant and susceptible categories showed higher endophytic colonization, greater diversity and more pathogen antagonistic organisms in the former (Feng et al., 2013). Studies with other plant systems have also suggested the prevalence of a similar relationship (Araújo et al., 2002; Reiter et al., 2002). The endophytic communities perhaps are not random guests but essential associates interacting with the hosts (Gaiero et al., 2013; Podolich et al., 2015). It is postulated that the endophytic bacteria, which are largely in non-cultivable form, perhaps play an active role in crop protection through their revival to active form in response to pathogen attack or environmental stress (Podolich et al., 2015).

It was significant to note that several of the endophytes from ^R^Arka Abha were positive for biocontrol properties compared to ^S^Arka Vikas. The promising antagonistic organisms *P. oleovorans* and *P. ananatis* were siderophore producers while *E. cloacae* and* P. ananatis* showed HCN production indicating a relationship between antagonistic ability and siderophore/HCN production. On the other hand, no clear relationship between antibiotic (NRPS/PKS) biosynthesis capability and antagonistic property was observed. Therefore, it was imperative to undertake direct pathogen challenge assays to determine the antagonistic potential of the organisms.

Past investigations that reported elucidation of wilt-disease resistance mechanisms against *R. solanacearum* often laid emphasis on tissue-structural (Rahman and Abdullah, 1997; Rahman et al., 1999), genetic (Wang et al., 2000; Yang and Francis, 2006), or molecular attributes (Jacobs et al., 2012; Coll and Valls, 2013). It is generally concluded that the resistance trait of different cultivars is under genetic control. A perusal of reports on genetic basis of *Ralstonia* wilt resistance in tomato, however, showed considerable variations in the inheritance of this trait depending on the test hybrid combinations or the pathogen-isolate employed. This varied from monogenic to digenic dominant or recessive, or polygenic inheritance (Grimault et al., 1995; Mohamed et al., 1997; Hanson et al., 1998). The resistant cultivars have shown considerably low internal colonization by this pathogen than susceptible genotypes (Grimault et al., 1994; Rahman and Abdullah, 1997). The observations documented in this study raise a query whether the bacterial endophytes play either a direct active part or a supportive role in governing the resistance feature of a cultivar synergistic with the current concept of genetic inheritance of resistance.

Generally it is believed that the endophytes are recruited from the soil environment by the host influenced by the soil type where the host genotype is also known to have a significant influence (Compant et al., 2010; Lundberg et al., 2012; Mueller et al., 2015). It is difficult to visualize selective acquisition/recruitment of endophytes to take place from the soil in a resistant cultivar. The present study in which the seedlings were grown in pasteurized cocopeat ensured to be devoid of pathogenic *Ralstonia* leaves no room for such selective recruitment. The host genotype is known to play a significant role in governing the plant associated microorganisms, particularly endophytes (Hartmann et al., 2009; Lundberg et al., 2012; Bakker et al., 2013; Podolich et al., 2015). There are also reports on transmission of endophytes through seeds (Hardoim et al., 2012; Truyens et al., 2014) which would explain their possible integral association with a particular host cultivar. This study, supported by the recent reports on intracellular colonization by bacterial endophytes (Thomas and Reddy, 2013; Thomas and Sekhar, 2014), suggests the possibility of maternal inheritance of endophytes as seed colonizers. This hypothesis necessitates the isolation of same organisms from different batches of a cultivar. A subsequent trial with ^S^Arka Vikas showed three of the five isolates same as the earlier set (*P. oleovorans, A. tumefaciens,* and *Microbacterium* sp.) while two isolates constituted different organisms (*Mitsuaria chitosanitabida* and *Kocuria palustris*) indicating vertical transmission as well as lateral recruitment of bacterial endophytes. Three repeat trials with ^R^Arka Abha showed antagonistic *P. oleovorans* as a common associate. The current opinion on seed-transmission of endophytes appears divided with some in favor while others remaining inconclusive (Hallmann, 2001; Hardoim et al., 2012; Truyens et al., 2014). It now calls for more detailed investigations on seed colonization and vertical transmission of endophytes *vis-à-vis* genetic control of disease resistance. Observations with aseptically grown watermelon (Thomas and Aswath, 2014) and preliminary observations with papaya *in vitro* systems (Thomas, unpublished data) endorsed this possibility.

In this study, our main objective was to understand if the native endophytes in different tomato genotypes had any bearing on the inherent resistance characteristic of a cultivar. This study was confined to the natural endophytes without any external fortifications. It needs further investigations to elucidate how the organisms protect the crop in natural conditions; whether they act singly or synergistically, and their interactive action with other rhizospheric organisms. For instance, *P. oleovorans* constituted the most common endophyte in Arka Vikas, but this cultivar was susceptible to the pathogen (Thomas et al., 2015). It is possible that the population level of this antagonist in ^S^Arka Vikas was low to offer any formidable protection against the pathogenic intruder. It is feasible to increase the population levels of this endophyte through seed/seedling fortification which perhaps may impart some pathogen resistance in this cultivar. There is a general criticism that the *in vitro* antagonism activity by the endophytes may not be translated into effective biocontrol strategies. Our preliminary trials also suggested that exploiting antagonistic agents as potential biocontrol agents has uncertain results. The biocontrol effects are influenced by various other factors. The significance of microbe–microbe interactions in antimicrobial activity among soil bacteria is being increasingly recognized now (Tyc et al., 2014). Therefore, additional trials are needed to work out the biocontrol strategy which forms the next action plan.

In this study, the identification of the organisms was determined based on 16S rRNA sequence homology to the sequences available at the NCBI GenBank and RDP databases, and the final identity was fixed as per the genus/species assigned by the GenBank. The identification of some of the organisms based on such single gene data may not be conclusive as demonstrated with *Pseudomonas* spp. (Hilario et al., 2004). Classification based on additional genes is envisaged as we proceed with the biocontrol studies in the case of promising organisms.

The isolates from ^R^Arka Abha (*P. oleovorans, P. ananatis,* and *E. cloacae*) which showed strong antagonistic activity and that from ^S^Arka Vikas (*P. oleovorans*) are now short listed for further biocontrol investigations. The two isolates of *P. oleovorans* (Tm-Av01 and Tm-Ab01) and one *A. tumefaciens* isolate (Tm-Ab09) also showed higher seedling vigor index over uninoculated control in both tomato cultivars offering scope for their exploitation in organic vegetable growing (Thomas and Upreti, 2015). The hallmark of this study has been the elucidation that the native endophytic bacterial floras associated with the seedlings in resistant cultivars perhaps play a role in natural defense against the pathogen which hypothesis goes synergistic with the current concept of genetic inheritance of disease resistance. The present findings contribute to a better understanding of the basic aspects related to host - pathogen - endophyte interactions and open the scope for further explorations into the biological control of this pathogen.

## Author Contributions

The experiments were planned together by the two authors. Bacterial isolation, PCR, and antagonistic assays were undertaken by RU. Bacterial identification, data interpretation, and manuscript preparation were done by PT. This work forms a part of the doctoral thesis of RU. The publication bears the Institute Contribution No. IIHR 92/2014.

## Conflict of Interest Statement

The authors declare that the research was conducted in the absence of any commercial or financial relationships that could be construed as a potential conflict of interest.

## References

[B1] Achari G. A. Ramesh R. (2014). Diversity, biocontrol, and plant growth promoting abilities of xylem residing bacteria from solanaceous crops. *Int. J. Microbiol.* 2014:14 10.1155/2014/296521 PMC4055287 24963298

[B2] Ahmad F. Ahmad I. Khan M. S. (2008). Screening of free living rhizospheric bacteria for their multiple plant growth promoting activities. *Microbiol. Res.* 163 173–181 10.1016/j.micres.2006.04.001 16735107

[B3] Araújo W. L. Marcon J. Maccheroni W. van Elsas J. D. van Vuurde J. W. Azevedo J. L. (2002). Diversity of endophytic bacterial populations and their interaction with *Xylella fastidiosa* in citrus plants. *Appl. Environ. Microbiol.* 68 4906–4914 10.1128/AEM.68.10.4906-4914.2002 12324338 PMC126398

[B4] Bakker P. A. H. M. Berendsen R. L. Doornbos R. F. Wintermans P. C. A. Pieterse C. M. J. (2013). The rhizosphere revisited: root microbiomics. *Front. Plant Sci.* 4:165 10.3389/fpls.2013.00165 PMC3667247 23755059

[B5] Coll N. S. Valls M. (2013). Current knowledge on the *Ralstonia solanacearum* type III secretion system. *Microb. Biotechnol.* 6 614–620. 23617636 10.1111/1751-7915.12056 PMC3815929

[B6] Compant S. Clément C. Sessitsch A. (2010). Plant growth-promoting bacteria in the rhizo- and endosphere of plants: their role, colonization, mechanisms involved and prospects for utilization. *Soil Biol. Biochem.* 42 669–678 10.1016/j.soilbio.2009.11.024

[B7] Compant S. Duffy B. Nowak J. Clément C. Barka E. A. (2005). Use of plant growth-promoting bacteria for biocontrol of plant diseases: principles, mechanisms of action, and future prospects. *Appl. Environ. Microbiol.* 71 4951–4959 10.1128/AEM.71.9.4951-4959.2005 16151072 PMC1214602

[B8] Compant S. Mitter B. Colli-Mull J. G. Gangl H. Sessitsch A. (2011). Endophytes of grapevine flowers, berries and seeds: identification of cultivable bacteria, comparison with other plant parts, and visualization of niches of colonization. *Microb. Ecol.* 62 188–197 10.1007/s00248-011-9883-y 21625971

[B9] Conn V. M. Walker A. R. Franco C. M. M. (2008). Endophytic actinobacteria induce defense pathways in *Arabidopsis thaliana*. *Mol. Plant Microb. Interact.* 21 208–218 10.1094/MPMI-21-2-0208 18184065

[B10] de Almeida C. V. Andreote F. D. Yara R. Tanaka F. A. O. Azevedo J. L. de Almeida M. (2009). Bacteriosomes in axenic plants: endophytes as stable endosymbionts. *World J. Microbiol. Biotech.* 25 1757–1764 10.1007/s11274-009-0073-8

[B11] Feng H. Li Y. Liu Q. (2013). Endophytic bacterial communities in tomato plants with differential resistance to *Ralstonia solanacearum*. *Afr. J. Microbiol. Res.* 7 1311–1318.

[B12] Gaiero J. R. McCall C. A. Thompson K. A. Day N. J. Best A. S. Dunfield K. E. (2013). Inside the root microbiome: bacterial root endophytes and plant growth promotion. *Am. J. Bot.* 100 1738–1750 10.3732/ajb.1200572 23935113

[B13] Genin S. Denny T. P. (2012). Pathogenomics of the *Ralstonia solanacearum* species complex. *Annu. Rev. Phytopathol.* 50 67–89 10.1146/annurev-phyto-081211-173000 22559068

[B14] Gómez-Lama Cabanás C. Schilirò E. Valverde-Corredor A. Mercado-Blanco J. (2014). The biocontrol endophytic bacterium *Pseudomonas fluorescens* PICF7 induces systemic defense responses in aerial tissues upon colonization of olive roots. *Front. Microbiol.* 5:427 10.3389/fmicb.2014.00427 PMC4155815 25250017

[B15] Grimault V. Anais G. Prior P. (1994). Distribution of *Pseudomonas solanacearum* in the stem tissues of tomato plants with different levels of resistance to bacterial wilt. *Plant Pathol.* 43 663–668 10.1111/j.1365-3059.1994.tb01604.x

[B16] Grimault V. Prior P. Anais G. (1995). A monogenic dominant resistance of tomato to bacterial wilt in Hawaii 7996 is associated with plant colonization by *Pseudomonas solanacearum*. *J. Phytopathol.* 143 349–352 10.1111/j.1439-0434.1995.tb00274.x

[B17] Hallmann J. (2001). “Plant interactions with endophytic bacteria,” in *Biotic Interactions in Plant-Pathogen Associations* eds Jeger M. J. Spence N. J. (Wallingford, Oxon: CABI Publishing), 87–119 10.1079/9780851995120.0087

[B18] Hallmann J. Quadt-Hallmann A. Mahaffe W. F. Kloepper J. W. (1997). Bacterial endophytes in agricultural crops. *Can. J. Microbiol.* 43 895–914 10.1139/m97-131

[B19] Hanson P. M. Licardo O. Wang J. F. Chen J. T. (1998). Diallel analysis of bacterial wilt resistance in tomato derived from different sources. *Plant Dis.* 82 74–78 10.1094/PDIS.1998.82.1.74 30857073

[B20] Hardoim P. R. Hardoim C. C. P. van Overbeek L. S. van Elsas J. D. (2012). Dynamics of seed-borne rice endophytes on early plant growth stages. *PLoS ONE* 7:e30438 10.1371/journal.pone.0030438 PMC3281832 22363438

[B21] Hartmann A. Schmid M. van Tuinen D. Berg G. (2009). Plant-driven selection of microbes. *Plant Soil* 321 235–257 10.1007/s11104-008-9814-y

[B22] Hayward A. C. (1991). Biology and epidemiology of bacterial wilt caused by *Pseudomonas solanacearum*. *Annu. Rev. Phytopathol.* 29 65–87 10.1146/annurev.py.29.090191.000433 18479193

[B23] Hilario E. Buckley T. R. Young J. M. (2004). Improved resolution on the phylogenetic relationships among *Pseudomonas* by the combined analysis of atpD, carA, recA and 16S rDNA. *Antonie Van Leeuwenhoek* 86 51–64 10.1023/B:ANTO.0000024910.57117.16 15103237

[B24] Jacobs J. M. Babujee L. Meng F. Milling A. Allen C. (2012). The in planta transcriptome of *Ralstonia solanacearum*: conserved physiological and virulence strategies during bacterial wilt of tomato. *MBio* 3:e00114-12 10.1128/mBio.00114-12 PMC3413399 22807564

[B25] Kelman A. (1954). The relationship of pathogenicity in *Pseudomonas solanacearum* to colony appearance on a tetrazolium medium. *Phytopathology* 44 693–696.

[B26] Lundberg D. S. Lebeis S. L. Paredes S. H. Yourstone S. Gehring J. Malfatti S. (2012). Defining the core *Arabidopsis thaliana* root microbiome. *Nature* 488 86–90 10.1038/nature11237 22859206 PMC4074413

[B27] Mansfield J. Genin S. Magori S. Citovsky V. Sriariyanum M. Ronald P. (2012). Top 10 plant pathogenic bacteria in molecular plant pathology. *Mol. Plant Pathol.* 13 614–629 10.1111/j.1364-3703.2012.00804.x 22672649 PMC6638704

[B28] Mercado-Blanco J. Lugtenberg B. J. J. (2014). Biotechnological applications of bacterial endophytes. *Curr. Biotechnol.* 3 60–75 10.2174/22115501113026660038

[B29] Mercado-Blanco J. Prieto P. (2012). Bacterial endophytes and root hairs. *Plant Soil* 361 301–306 10.1007/s11104-012-1212-9

[B30] Miller K. I. Qing C. Sze D. M. Y. Neilan B. A. (2012). Investigations of the biosynthetic potential of endophytes in traditional Chinese anticancer herbs. *PLoS ONE* 7:e35953 10.1371/journal.pone.0035953 PMC3358349 22629306

[B31] Mohamed M. E. S. Umaharan P. Phelps R. H. (1997). Genetic nature of bacterial wilt resistance in tomato (*Lycopersicon esculentum* Mill.) accession La 1421. *Euphytica* 96 323–326 10.1023/A:1003075427304

[B32] Mueller H. Berg C. Landa B. B. Auerbach A. Moissl-Eichinger C. Berg G. (2015). Plant genotype-specific archaeal and bacterial endophytes but similar *Bacillus* antagonists colonize Mediterranean olive trees. *Front. Microbiol.* 6:138 10.3389/fmicb.2015.00138 PMC4347506 25784898

[B33] Pirttilä A. M. Laukkanen H. Pospiech H. Myllyla R. Hohtola A. (2000). Detection of intracellular bacteria in the buds of Scotch pine (*Pinus sylvestris* L.) by in situ hybridization. *Appl. Environ. Microbiol*. 66 3073–3077 10.1128/AEM.66.7.3073-3077.2000 10877808 PMC92113

[B34] Podolich O. Ardanov P. Zaets I. Pirttilä A. M. Kozyrovska N. (2015). Reviving of the endophytic bacterial community as a putative mechanism of plant resistance. *Plant Soil* 388 367–377 10.1007/s11104-014-2235-1

[B35] Prieto P. Schilirò E. Maldonado-González M. M. Valderrama R. Barroso-Albarracín J. B. Mercado-Blanco J. (2011). Root hairs play a key role in the endophytic colonization of olive roots by *Pseudomonas* spp. with biocontrol activity. *Microb. Ecol.* 62 435–445 10.1007/s00248-011-9827-6 21347721 PMC3155037

[B36] Rahman M. A. Abdullah H. (1997). Susceptibility of *Capsicum* species and cultivars to *Ralstonia solanacearum*: anatomical differences and bacterial multiplication in resistant and susceptible cultivars. *Pertanika J. Trop. Agric. Sci.* 20 1–11.

[B37] Rahman M. A. Abdullah H. Vanhaecke M. (1999). Histopathology of susceptible and resistant *Capsicum annuum* cultivars infected with *Ralstonia solanacearum*. *J. Phytopathol.* 147 129–140 10.1111/j.1439-0434.1999.tb03819.x

[B38] Ramesh R. Phadke G. S. (2012). Rhizosphere and endophytic bacteria for the suppression of eggplant wilt caused by *Ralstonia solanacearum*. *Crop Prot.* 37 35–41 10.1016/j.cropro.2012.02.008

[B39] Reinhold-Hurek B. Krause A. Leyser B. Miché L. Hurek T. (2007). “The rice apoplast as a habitat for endophytic N2-fixing bacteria,” in *The Apoplast of Higher Plants: Compartment of Storage, Transport and Reactions*, eds Sattelmacher B. Horst W. J. (Berlin: Springer), 427–443 10.1007/978-1-4020-5843-1_30

[B40] Reiter B. Pfeifer U. Schwab H. Sessitsch A. (2002). Response of endophytic bacterial communities in potato plants to infection with *Erwinia carotovora* subsp. atroseptica. *Appl. Environ. Microbiol.* 68 2261–2268 10.1128/AEM.68.5.2261-2268.2002 11976096 PMC127529

[B41] Ryan R. P. Germaine K. Franks A. Ryan D. J. Dowling D. N. (2008). Bacterial endophytes: recent development and applications. *FEMS Microbiol. Lett.* 278 1–9 10.1111/j.1574-6968.2007.00918.x 18034833

[B42] Sattelmacher B. (2001). The apoplast and its significance for plant mineral nutrition. *New Phytol.* 149 167–192 10.1046/j.1469-8137.2001.00034.x 33874640

[B43] Schwyn B. Neilands J. B. (1987). Universal chemical assay for the detection and determination of siderophores. *Anal. Biochem.* 160 47–56 10.1016/0003-2697(87)90612-9 2952030

[B44] Sessitsch A. Hardoim P. Döring J. Weilharter A. Krause A. Woyke T. (2012). Functional characteristics of an endophyte community colonizing rice roots as revealed by metagenomic analysis. *Mol. Plant Microb. Interact.* 25 28–36 10.1094/MPMI-08-11-0204 21970692

[B45] Tan H. Zhou S. Deng Z. He M. Cao L. (2011). Ribosomal-sequence-directed selection for endophytic streptomycete strains antagonistic to *Ralstonia solanacearum* to control tomato bacterial wilt. *Biol. Control* 59 245–254 10.1016/j.biocontrol.2011.07.018

[B46] Ting A. S. Y. (2014). “Biosourcing endophytes as biocontrol agents of wilt diseases,” in *Advances in Endophytic Research*, eds Verma V. C. Gange A. C. (New Delhi: Springer), 283–300.

[B47] Thomas P. Aswath C. (2014). In vitro introduction of hardy alcohol resistant *Bacillus* spp. through aseptically grown watermelon seedlings. *Adv. Microbiol*. 4 504–510 10.4236/aim.2014.49056

[B48] Thomas P. Reddy M. K. (2013). Microscopic elucidation of abundant endophytic bacteria colonizing the cell wall – plasma membrane peri-space in the shoot-tip tissue of banana. *AoB Plants* 5:plt011 10.1093/aobpla/plt011

[B49] Thomas P. Sadashiva A. T. Upreti R. Mujawar M. M. (2015). Direct delivery of inoculum to shoot tissue interferes with genotypic resistance to *Ralstonia solanacearum* in tomato seedlings. *J. Phytopathol.* 163 320–323 10.1111/jph.12281

[B50] Thomas P. Sekhar A. C. (2014). Live cell imaging reveals extensive intracellular cytoplasmic colonization of banana genotypes by normally non-cultivable endophytic bacteria. *AoB Plants* 6:plu002 10.1093/aobpla/plu002 PMC4038436 24790123

[B51] Thomas P. Sekhar A. C. Mujawar M. M. (2012). Non-recovery of varying proportions of viable bacteria during spread-plating governed by the extent of spreader usage and proposal for an alternate spotting-spreading approach to maximize the CFU. *J. Appl. Microbiol.* 113 339–350 10.1111/j.1365-2672.2012.05327.x 22563785

[B52] Thomas P. Swarna G. K. Roy P. K. Patil P. (2008). Identification of cultivable and originally non-culturable endophytic bacteria isolated from shoot tip cultures of banana cv. Grand Naine. *Plant Cell Tiss. Org. Cult.* 93 55–63 10.1007/s11240-008-9341-9

[B53] Thomas P. Upreti R. (2014a). Testing of bacterial endophytes from non-host sources as potential antagonistic agents against tomato wilt pathogen *Ralstonia solanacearum*. *Adv. Microbiol.* 4:656–666 10.4236/aim.2014.410071

[B54] Thomas P. Upreti R. (2014b). Influence of seedling age on the susceptibility of tomato plants to *Ralstonia solanacearum* during protray screening and at transplanting. *Am. J. Plant Sci.* 5 1755–1762 10.4236/ajps.2014.512190

[B55] Thomas P. Upreti R. (2014c). Significant effects due to peptone in Kelman medium on colony characteristics and virulence of *Ralstonia solanacearum* in tomato. *Open J. Microbiol.* 8 87–105 10.2174/1874285801408010095 PMC4235106 25408775

[B56] Thomas P. Upreti R. (2015). Evaluation of tomato seedling-root associated bacterial endophytes towards organic seedling production *Org. Agri.* 10.1007/s13165-015-0111-9

[B57] Truyens S. Weyens N. Cuypers A. Vangronsveld J. (2014). Bacterial seed endophytes: genera, vertical transmission and interaction with plants. *Environ. Microbiol. Rep*. 7 40–50 10.1111/1758-2229.12181

[B58] Turner T. R. James E. K. Poole P. S. (2013). The plant microbiome. *Genome Biol.* 14:209 10.1186/gb-2013-14-6-209 PMC3706808 23805896

[B59] Tyc O. van den Berg M. Gerards S. van Veen J. A. Raaijmakers J. M. de Boer W. Garbeva P. (2014). Impact of interspecific interactions on antimicrobial activity among soil bacteria. *Front. Microbiol.* 5:567 10.3389/fmicb.2014.00567 PMC4211544 25389421

[B60] Vanitha S. C. Niranjana S. R. Mortensen C. N. Umesha S. (2009). Bacterial wilt of tomato in Karnataka and its management by *Pseudomonas fluorescens*. *Biocontrol* 54 685–695 10.1007/s10526-009-9217-x

[B61] Wang J. F. Olivier J. Thoquet P. Mangin B. Sauviac L. Grimsley N. H. (2000). Resistance of tomato line Hawaii7996 to *Ralstonia solanacearum* Pss4 in Taiwan is controlled mainly by a major strain-specific locus. *Mol. Plant Microb. Interact.* 13 6–13 10.1094/MPMI.2000.13.1.6 10656580

[B62] Yang W. Francis D. M. (2006). “Genetics and breeding for bacterial diseases in tomato: prospects for marker-assisted selection,” in *Genetic Improvement of Solanaceous Crops* Vol. 2* Tomato,* eds Razdan M. J. Mattoo A. K. (Boca Raton, FL: CRC Press), 381–414.

[B63] Zinniel D. K. Lambrecht P. Harris N. B. (2002). Isolation and characterization of endophytic colonizing bacteria from agronomic crops and prairie plants. *Appl. Environ. Microbiol*. 68 2198–2208 10.1128/AEM.68.5.2198-2208.2002 11976089 PMC127535

